# Magnetically Controlled Hyaluronic Acid–Maghemite Nanocomposites with Embedded Doxorubicin

**DOI:** 10.3390/polym15173644

**Published:** 2023-09-04

**Authors:** Vasily Spiridonov, Zukhra Zoirova, Yuliya Alyokhina, Nikolai Perov, Mikhail Afanasov, Denis Pozdyshev, Daria Krjukova, Alexander Knotko, Vladimir Muronetz, Alexander Yaroslavov

**Affiliations:** 1 Department of Chemistry, Lomonosov Moscow State University, Leninskie Gory 1-3, 119991 Moscow, Russia; 2 Faculty of Materials Science, Lomonosov Moscow State University, Leninskie Gory 1-73, 119991 Moscow, Russia; 3 Department of Physics, Lomonosov Moscow State University, Leninskie Gory 1-2, 119991 Moscow, Russia; 4 Belozersky Research Institute of Physico-Chemical Biology, Lomonosov Moscow State University, Leninskye gory 1-40, 119992 Moscow, Russiavimuronets@mail.ru (V.M.)

**Keywords:** magnetic polymer–iron oxide composites, biopolymer, enzyme, biodegradability, cytotoxicity

## Abstract

The controllable delivery of drugs is a key task of pharmacology. For this purpose, a series of polymer composites was synthesized via the cross-linking of hyaluronate and a hyaluronate/polyacrylate mixture with Fe_2_O_3_ nanoparticles. The cross-linking imparts magnetic properties to the composites, which are more pronounced for the ternary hyaluronate/polyacrylate/γ-Fe_2_O_3_ composites compared with the binary hyaluronate/Fe_2_O_3_ composites. When dispersed in water, the composites produce microsized hydrogel particles. Circulation of the ternary microgels in an aqueous solution at a speed of 1.84 cm/s can be stopped using a permanent external magnet with a magnetic flux density of 400 T. The composite hydrogels can absorb the antitumor antibiotic doxorubicin (Dox); the resulting constructs show their cytotoxicity to tumor cells to be comparable to the cytotoxicity of Dox itself. The addition of the hyaluronidase enzyme induces degradation of the binary and ternary microgels down to smaller particles. This study presents prospectives for the preparation of magnetically controlled biodegradable polymer carriers for the encapsulation of bioactive substances.

## 1. Introduction

Magnetically controlled polymeric conjugates have been shown to be effective drug carriers [1,2]. Being sensitive to an external magnetic field, the conjugate–drug complex can be delivered to a specific organ to be treated [3,4]. The targeted delivery of the drug results in reducing the therapeutic dose, eliminating side effects and smoothing fluctuations in the circulating drug level [5,6]. It has been reported that the heating of tumor cells, using heating of accumulated magnetic conjugates in AC magnetic field, above 42 °C induces a local thermal destruction of the tumor [7,8]. Chemotherapy combined with hyperthermia enhances the efficacy of anticancer treatment [9,10]. This combination allows for invasive and safe therapy, while the antitumor efficacy is significantly increased compared with the efficacy of chemotherapy alone [11,12].

When constructing the conjugates, nanosized particles of iron oxides are widely used because of their high magnetic susceptibility and low toxicity [13,14]. In order to stabilize the magnetic nanoparticles against aggregation, they are embedded into a water-soluble anionic polymer matrix, for example, synthetic polyacrylic acid or native polysaccharides [15,16].

Biomedical applications require stabilized magnetic polymer conjugates with certain fundamental and operational characteristics, including a specific size which controls the magnetic properties of the conjugates [17], the total negative charge of particles responsible for their colloidal stability in a biological environment [18], the sufficient capacity of particles to encapsulate the drug [19], negligible toxicity [20] and the ability of the particles to biodegrade after completing their transport function [21]. Following these requirements, magnetic polymer formulations have been synthesized for magnetic resonance imaging [22], the separation of mammalian and bacterial cells [23,24], genetic engineering [25] and medical diagnostics and treatments [26,27,28,29]. 

In the present article, we describe the synthesis of microsized composites from sodium hyaluronate or a hyaluronate/polyacrylate mixture, cross-linked with maghemite nanoparticles; the physicochemical properties of the incorporated maghemite nanoparticles; and the composition and hydrodynamic and electrokinetic properties of polymer-nanoparticle hydrogels. Special attention is paid to the ability of an external magnetic field to stop the movement of hydrogels in an aqueous solution and concentrate them on the capillary wall. Additionally, the incorporation of antitumor antibiotic doxorubicin in the polymer matrix, the cytotoxicity of the resulting polymer–Dox constructs, the enzymatic cleavage of the polymer matrix and the release of Dox are discussed. The results are of importance for constructing biodegradable carriers with controllable magnetic properties.

## 2. Experimental Section

### 2.1. Materials

Mohr’s salt (NH_4_)_2_Fe(SO_4_)_2_ × 5H_2_O) (Chimmed, Moscow, Russia), NaH_2_PO_2_ (VK Labor und Feinchemikalien, Dresden, Germany), NaOH (Chemapol, Praha, Czech Republic), doxorubicin hydrochloride (Teva, Israel), sulfosalicylic acid and sodium polyacrylate (PA) with Mw = 90 kDa (both from Merck, Rahway, NJ, USA) were used as received. Sodium hyaluronate (HYAL) with Mw = 250 kDa (Riedel-deHaen, Czech Republic) was dialyzed against bi-distilled water for three days and then lyophilized. 

The magnetic polymeric composites were obtained via the one-step synthesis of 𝛾-Fe_2_O_3_ from Mohr’s salt in the presence of HYAL [30]. A total of 12.9 ÷ 50 mg of Mohr’s salt was dispersed in 1 mL of distilled water and added dropwise to 5 mL of 0.5 wt% HYAL water solution. Then, 1 mL of water solution with 1.5 ÷ 10.5 mg of NaOH and 1 mL of water solution with 5.28 ÷ 30.3 mg of NaH_2_PO_2_ were added, and the resulting solution was stirred for 24 h. A specific amount of reagent was taken depending on what composite was to be obtained. The reaction was completed when the green color of the initial mixture changed to a dark color. The resulting solutions were dialyzed against bi-distilled water for three days and then lyophilized to produce the magnetic HYAL-γ-Fe_2_O_3_ composites. The content of iron in the samples (η) was quantified via spectrophotometric titration with sulfosalicylic acid [31] as described earlier.

In order to prepare hybrid magnetic composites with native HYAL and synthetic PA, 5 ÷ 25 mg of Mohr’s salt was dispersed in 1 mL of distilled water and added dropwise to 10 mL of a water solution with 10 mg of HYAL and 0.5 ÷ 3 mg of PA. Then, 1 mL of water solution with 1.5 ÷ 10.5 mg of NaOH and 1 mL of water solution with 5.28 ÷ 30.3 mg of NaH_2_PO_2_ were added, and the resulting solution was stirred for 24 h. A specific amount of reagent was taken depending on what composite was to be obtained. The reaction was completed when the green color of the initial mixture changed to a dark color. The resulting solutions were dialyzed against bi-distilled water for three days and then lyophilized to produce the hybrid magnetic HYAL-PA-γ-Fe_2_O_3_ composites. 

Encapsulation of Dox into the polymer-γ-Fe_2_O_3_ composites was performed as described earlier [32]. Briefly, aqueous solutions of HYAL or HYAL/PA mixture were mixed with an aqueous Dox solution. The resulting solutions were stirred for 24 h, then dialyzed against bi-distilled water for three days and lyophilized to produce the magnetic binary HYAL-γ-Fe_2_O_3_-Dox and ternary HYAL/PA-γ-Fe_2_O_3_-Dox composites. The composites were dispersed in a pH 7.2 TRIS buffer solution. Optical densities of solutions, measured spectrophotometrically at 580 nm, showed that more than 99% of the initial Dox was involved in the magnetic composites. 

### 2.2. Methods

Phase identification of 𝛾-Fe_2_O_3_ nanoparticles in the dried hydrogel was performed with X-ray diffraction using D/MAX 2500 diffractometer (Rigaku, Tokyo, Japan). 

Mössbauer spectra were recorded using an MS-1104EM (Chernogolovka, Russia) spectrometer with ^57^Co (Rh) sources. The absorber thickness was 0.12 mg/cm^2^ (57Fe). The values of isomeric shifts are presented relative to α-Fe. 

The content of iron in the composite was determined spectrophotometrically with an Ultrospec 4050 unit (LKB, Kiruna, Sweden). The composite (5 mg) was dissolved in 10 μL of concentrated H_2_SO_4_, and then a 10% solution of sulfosalicylic acid (10 mL) was added. The resulting solution was adjusted to pH 2. The optical density was measured at λ = 510 nm taking the extinction coefficient of ε = 1800 L/(mol cm). The amount of iron in the sample was determined using the calibration plot.

The enzyme action on the polysaccharide component of magnetic microgels was determined spectrophotometrically using an Ultrospec 4050 unit (LKB, Kiruna, Sweden). For this purpose, 1 mg/mL solutions of microgels in Tris buffer with pH 7.4 containing 0.15 M NaCl were prepared. The hyaluronidase enzyme was added to the obtained solutions, maintaining an enzyme concentration of 1 μg/mL, and absorption spectra were recorded. The results are presented as the optical density at λ = 234 nm vs. time plots. The accuracy of UV/vis method is 5%.

The fluorescence intensity was measured using an F-4000 Hitachi fluorimeter with the excitation wavelength λ(exc.) = 480 nm. The accuracy of fluorescence method is 7%.

Magnetic properties of the samples were studied with a Lakeshore 7400 vibrating Sample magnetometer (Lake Shore Cryotronics, Carson, CA, USA) at room temperature in a field range of ±16 kOe. 

Electrophoretic mobility of particles was measured via laser microelectrophoresis in a thermostatic cell using a Brookhaven Zeta Plus instrument with the corresponding software. The accuracy of laser microelectrophoresis method is 5%. 

Mean hydrodynamic diameter of particles was determined via dynamic light scattering (DLS) at a fixed scattering angle (90°) in a thermostatic cell with a Brookhaven Zeta Plus instrument (Holtsville, NY, USA). Software provided by the manufacturer was employed to calculate diameter values. 

Molecular mass characteristics of the polysaccharide and composites were measured using static light-scattering technique at a fixed scattering angle of 90°. The measurements were carried out using a Photocor Complex photometer (Photocor Instruments, Moscow, Russia) equipped with a He–Ne 10 mW laser (λ = 633 nm) as the light source at 25 °C. The results were processed using the Debye equation. The accuracy of light-scattering methods is 10%.

In order to visualize the initial anionic microgels and Fe-containing magnetic microgels, transmission electron microscopy (TEM) with a JEM-100B unit (JEML, Jem, Germany) was used. A drop of microgel aqueous solution was applied on a copper grid and dried. The samples were studied without preliminary contrasting. 

The size distribution of inorganic nanoparticles was estimated via manual counting of particles in the field of the microscope. The analysis of the number of particles was carried out using the Origin 9.0 program. 

The IR spectra were recorded using Specord M-80 Spectrophotometer (Carl Zeiss, Oberkochen, Germany) in the range of 2000–400 cm^−1^ at room temperature in KBr matrix. 

The cytotoxicity of the samples to human breast adenocarcinoma MCF-7 cells was assessed using the methylthiazolyl tetrazolium (MTT) assay [33]. The MCF-7 cells were obtained from a vertebrate cell culture collection (Institute of Cytology, Russian Academy of Sciences). Cells from the thawed vial were used in experiments for up to 10 passages. MCF-7 cells were cultured in DMEM/F12 medium (PanEco, Moscow, Russia), supplemented with 10% (*v*/*v*) fetal bovine serum (Hyclone, Logan, Utah, USA), 1% (*v*/*v*) L-glutamax (Sigma-Aldrich, St. Louis, MO, USA) and 1% (*v*/*v*) antibiotic solution (penicillin, streptomycin) (PanEco, Russia) and incubated at 37 °C in 5% CO_2_ and saturated humidity. For the cytotoxicity assay, the cells were seeded into a 96-well plate at a concentration of 3.8 × 10^3^ cells per well. After 24 h, the cells were incubated for 1 h with the medium alone or with a twofold serial dilution of tested samples, starting with the highest concentration at 10 µM. After 72 h of incubation in a complete medium, cell viability was estimated via the reduction of the MTT dye to formazan. For this, MTT solution (at a concentration of 0.375 mg/mL of medium) was added to each well and incubated for 4 h. The formazan crystals formed were dissolved in 100 µL of DMSO. After 15 min at 37 °C, the absorbance was registered at 570 nm using a microplate reader (VersaMax, San Jose, CA, USA). A reference wavelength of 630 nm was used. Cell viability was calculated from the absorbance ratio between the cell culture treated with the tested samples and the untreated control.

## 3. Results and Discussion

In this article, two types of magnetic polymeric composites are discussed, the first from sodium hyaluronate (HYAL) and the second from the sodium hyaluronate/polyacrylate (HYAL-PA) mixture, both cross-linked with maghemite nanoparticles. Below, each system is described in the following sequence: the formation of polymer–maghemite composites, analysis of their compositions and analysis of their physicochemical properties. In the special sections, the magnetic properties of the composites, their degradation by a specific enzyme and release of encapsulated Dox and the cytotoxicity of Dox-loaded composites are discussed. 

### 3.1. Synthesis of the Magnetic Binary Composites

Synthesis of the magnetic binary composites was conducted using a previously described protocol [16,30]. The magnetic Fe-containing particles were produced from Mohr’s salt in the presence of HYAL in an alkaline aqueous solution at room temperature. Varying the molar ratio of Mohr’s salt to the HYAL carboxyl group ([Fe(^2+^)]/[HYAL]) allowed for the preparation of composites with different iron contents (Table 1). The higher the [Fe^2+^]/[HYAL] ratio in a reaction mixture, the higher the iron content in the resulting composite (η).

**Figure 1:**
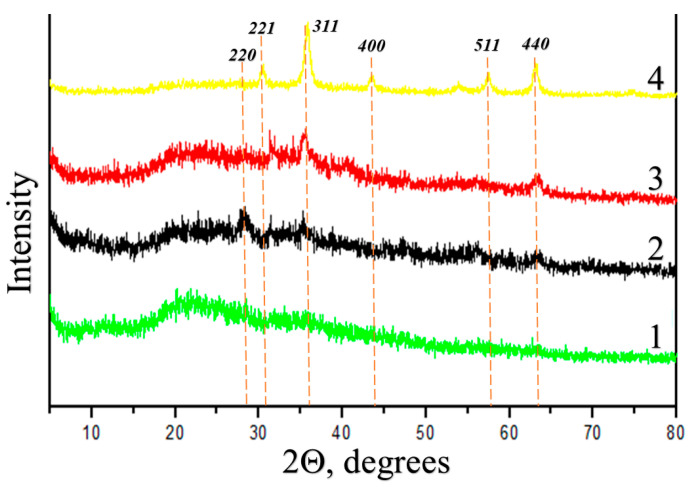
X-ray diffraction patterns of the binary composites I (1), II (2), III (3) and IV (4).

X-ray patterns of the four composites are shown in Figure 1. XRD analysis of the studied samples showed the single-phase nature of iron-containing nanoparticles [34,35]. In the XRD spectra of the synthesized samples, the positions of diffraction peaks (220), (221), (311), (400), (511) and (440) correspond to the cubic structure of spinel [36,37,38]. The strong peaks on the XRD spectra indicate the well-crystallized structure of the iron oxide phase [39].

However, in the obtained diffractograms one can observe both a significant broadening of peaks and their significant difference in intensity, which indicate the nanocrystalline character and polydispersity of the synthesized nanoparticles [40].

The above reflections, (220), (221), (311), (400), (511) and (440), correspond to iron oxides: magnetite FeO−Fe_2_O_3_, or maghemite γ-Fe_2_O_3_, or a magnetite/maghemite mixture [40]. In other words, the X-ray diffraction data are not sufficient to come to a definite conclusion about the chemical nature of inorganic inclusions. 

**Figure 2:**
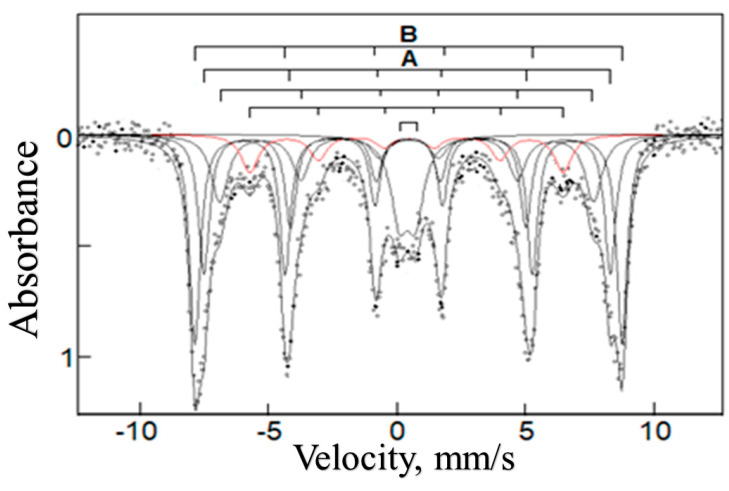
Mössbauer spectrum of the binary composite IV measured at 78 K.

The chemical structure of the inorganic particles was identified by using Mössbauer spectroscopy. The ^57^Fe Mössbauer spectrum of the Fe-containing composite IV with η = 26.8 wt% at 78 K shown in Figure 2 is a superposition of two sextets of the magnetic hyperfine structure and a quadrupole doublet. The sextets at the magnetic field H ≈ 490 and 515 kOe correspond to the A and B positions in the structure of γ-Fe_2_O_3_ particles [41]. Importantly, in the Mössbauer spectrum no sextet is observed for an “intermediate” oxidation state of Fe^2+^/Fe^3+^ (δ > 0.6 mm/s) [42], which clearly indicates the formation of only γ-Fe_2_O_3_ particles during the conjugate synthesis. Additionally, the Mössbauer spectrum allows the estimation of γ-Fe_2_O_3_ particle sizes: 7–10 nm. The quadrupole doublet evidences a fraction of small particles whose sizes are 4 nm and smaller [43,44,45,46,47].

**Figure 3:**
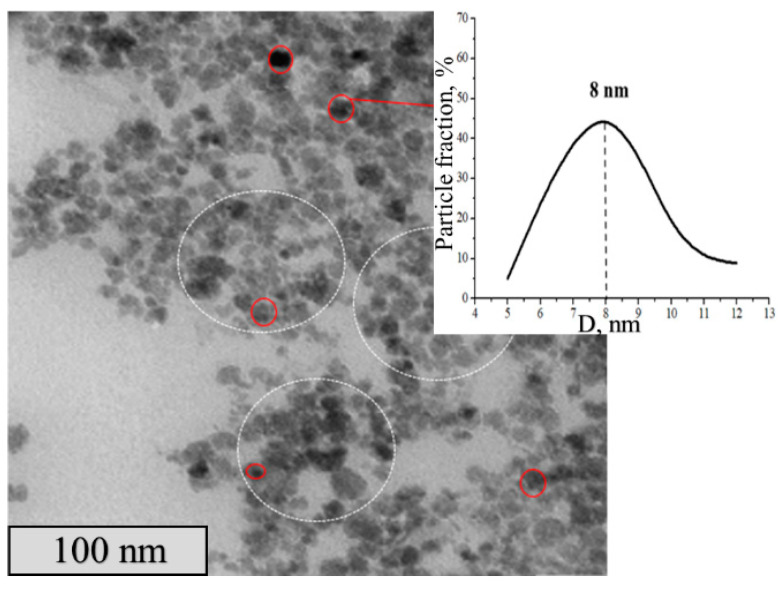
TEM image of the binary composite IV (**left**) and size distribution of the composite particles (**right**).

The γ- Fe_2_O_3_ nanoparticles in the composites were visualized with transmission electron microscopy. A typical picture (Figure 3) shows dark contrast nanoparticles, which correspond to Fe-containing inclusions, whose diameter lies within a 3–20 nm range with a mean size of 8 nm. These parameters, the mean size of γ- Fe_2_O_3_ nanoparticles and their size distribution, remain unchanged for all synthesized binary composites, from I to IV (see data for composites I–III in Supplementary Materials). 

**Figure 4:**
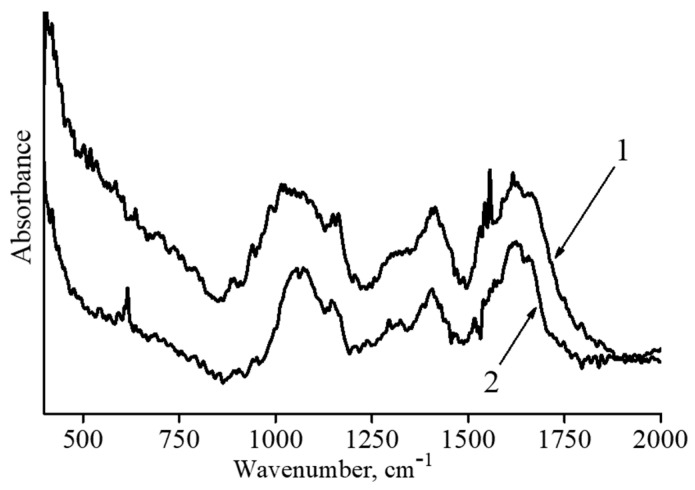
IR spectra of HYAL (1) and the binary composite IV (2).

The nature of interaction between the HYAL matrix and γ-Fe_2_O_3_ nanoparticles in the composites was examined with IR spectroscopy. The IR spectra for the initial HYAL and Fe-containing composite with the maximum iron oxide content η = 26.8 wt% are shown in Figure 4. 

In the initial HYAL spectrum (curve 1, Figure 4), there are peaks at 1680 cm^−1^ and 1620 cm^−1^ which relate to C=O vibrations of the amide I band and asymmetric vibrations of the carboxylate groups, respectively [48]. The absorption band (shoulder) at 1558 cm^−1^ is characteristic of the amide II band. The peak at 1158 cm^−1^ is assigned to the C–O−C group (O-bridge) [49], while the band at 900 cm^−1^ is assigned to an asymmetrical out-of-phase ring vibration [50]. 

An IR spectrum of the magnetic microgel (curve 2, Figure 4) retains peaks at 1680 cm^−1^ and 1558 cm^−1^ associated with the amide part. At the same time, a sharp decrease in the intensity of the 1620 cm^−1^ peak and a shift of the 1158 cm^−1^ peak are observed. These changes, together with the peak at 616 cm^−1^ corresponding to Fe–O vibrations, show that carboxyl groups and O-bridges apparently interact with the magnetic nanoparticle surface through the formation of electrostatic and coordination contacts.

All four synthesized composites were easily dispersed in bi-distilled water and aqueous–salt solutions, thus forming microgel particles. In both cases, dynamic light scattering (DLS) found one type of composite microgels with a narrow particle size distribution (see data for typical distribution functions in Supplementary Materials). The mean hydrodynamic diameters D*_h_* (sizes) of the composite microgels in bi-distilled water and a physiological 0.15 M NaCl solution are shown in Table 2. 

As follows from the data in Table 2, the sizes of the composite microgels progressively decreased with increasing iron oxide content. This was due to the contraction of HYAL macromolecules after their cross-linking with γ-Fe_2_O_3_ nanoparticles. The sizes of the microgels in the physiological solution were always smaller than their sizes in bi-distilled water. As expected, the low-molecular-weight salt shielded the negative charges of HYAL, which was accompanied by additional collapsing of the anionic macromolecules. 

The electrophoretic mobility (EPM) of the microgels (a parameter associated with the microgel surface charge) is shown in column 5, Table 2. Incorporation of γ-Fe_2_O_3_ nanoparticles into the HYAL matrix resulted in a partial neutralization of the HYAL charge; this effect was more pronounced for the microgels with higher γ-Fe_2_O_3_ content. 

Static light scattering (SLS) allowed the molecular mass characterization of the Fe-containing composites. According to conventional recommendations [51], the experiments were performed in an aqueous–salt solution with [NaCl] = 0.15 M, which suppressed repulsion between anionic HYAL groups and eliminated the effect of “polyelectrolyte swelling” when dissolving the composite microgels [52]. The weight average molecular masses (M_w_), calculated using Zimm diagrams, are summarized in Table 3 together with other hydrodynamic characteristics of the composite microgels: the radius of gyration R_g_, the hydrodynamic radius R*_h_*, R_g_/R*_h_* form factor and the second virial coefficient A_2_. 

A rise in the iron oxide content increased MW values from 100 × 10^6^ Da for composite I up to 250 × 10^6^ Da for composite IV (column 3, Table 3). This was due to the inclusion of more iron oxide nanoparticles and HYAL macromolecules into each composite species. 

The combination of DLS (column 4) and SLS (column 5) results gives a R_g_/R*_h_* form factor (column 6), a parameter that characterizes the geometry of scattering particles [53]. The R_g_/R*_h_* values from Table 3 are in the 0.44–0.64 range and, according to the literature [54,55], fall in the R_g_/R*_h_* interval typical for microgel particles (0.3–0.7). 

A second virial coefficient A_2_ is positive for the solutions of all four composites (column 7). This reflects the thermodynamic affinity of the Fe-containing binary microgels to a solvent, the aqueous–salt solution. 

To summarize, the HYAL-γ-Fe_2_O_3_ binary composites were synthesized with the Fe content varying from 5.1 up to 26.8 wt%. The sizes of the γ-Fe_2_O_3_ particles are in the 3–20 nm range with an average size of 8 nm. The composites are easily dispersed in water and aqueous–salt solutions, thus forming microsized hydrogels whose sizes are within a 150–275 nm range in bi-distilled water and a 110–160 nm range in a 0.15 M NaCl aqueous solution. The microgels carry a negative surface charge, which ensures their colloidal stability in aqueous media [56,57].

### 3.2. Synthesis of HYAL-PA-Maghemite Ternary Composites

Synthesis of Fe-containing HYAL-PA-maghemite ternary composites was carried out following to the above protocol, but modified so that 𝛾-Fe_2_O_3_ was formed in the presence of a HYAL-PA mixture. The quantification of the iron content η in four Fe-containing HYAL-PA ternary composites is reflected in Table 4. 

**Figure 5:**
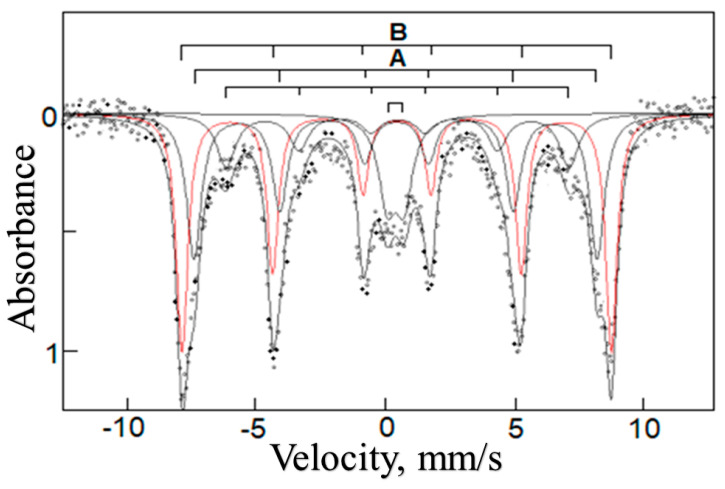
Mössbauer spectrum of the Fe-containing composite IVpa with η = 38 wt% measured at 78 K.

A typical Mössbauer spectrum of an Fe-containing ternary composite (a sample with η = 38 wt% was used) (Figure 5) is identical to the typical Mössbauer spectrum for an Fe-containing binary composite (Figure 2). This allows for conclusions about (1) γ-Fe_2_O_3_ particles in the ternary composite and (2) their size, which lies in the 7–10 nm region.

**Figure 6:**
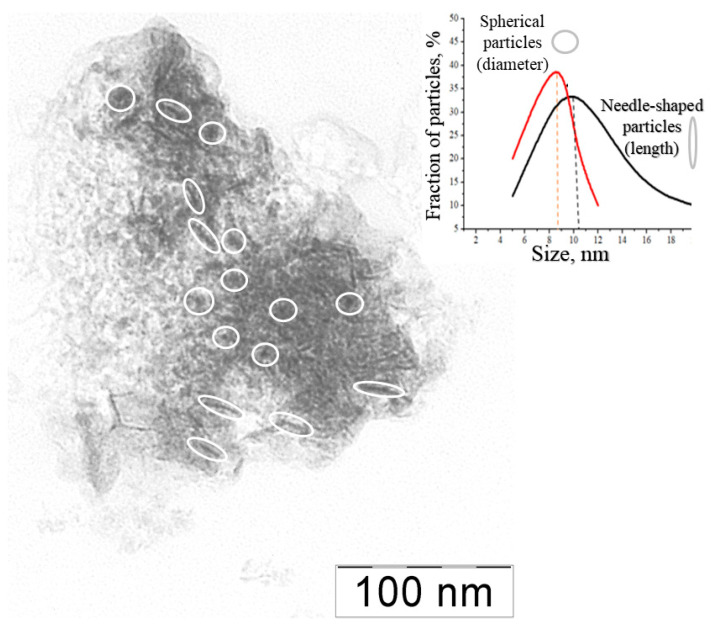
TEM image of the ternary composite IVpa (**left**) and size distribution of the composite IVpa particles (**right**).

The γ-Fe_2_O_3_ nanoparticles in the ternary composites were visualized with transmission electron microscopy. In a typical picture (Figure 6), there are two types of contrasting nanoparticles: the spherical ones with an average size of 10 nm and the needle-shaped ones with an average size of 12 nm. In general, the size of the particles falls in a 3–20 nm range that correlates with the estimation of their size using Mössbauer spectroscopy. 

Thus, with a previously described protocol, polymeric composites were synthesized in which a HYAL-PA mixed polymeric matrix was cross-linked by γ-Fe_2_O_3_ nanoparticles (whose size is in the 7–10 nm range). In this case, the nanoparticles have two different shapes. First, there are spherical particles with an average diameter near 9 nm and second, there are needle-shaped particles with an average length near 10 nm. The properties of the ternary samples largely repeat the properties of the HYAL- γ-Fe_2_O_3_ binary composites.

### 3.3. Magnetic Properties of the Polymer–Maghemite Composites

Magnetic properties are a key characteristic of the synthesized composites. They largely determine whether the composites can be used for controlled delivery of bioactive compounds. An applied external field should deliver the magnetically sensitive carrier to a certain place in the body and keep it there, sometimes for a long time, until the encapsulated substance is released and the therapeutic effect is manifested. The body’s natural way of transporting drugs is through the blood vessels. Taking this into account, the study of Fe-containing polymeric composites was carried out in two directions: the traditional model with solid composite samples, which allowed the quantifying of the magnetic properties of the composites, and the model in which the Fe-containing polymeric microgels moved in a capillary of a controlled cross section at a controlled speed. 

**Figure 7:**
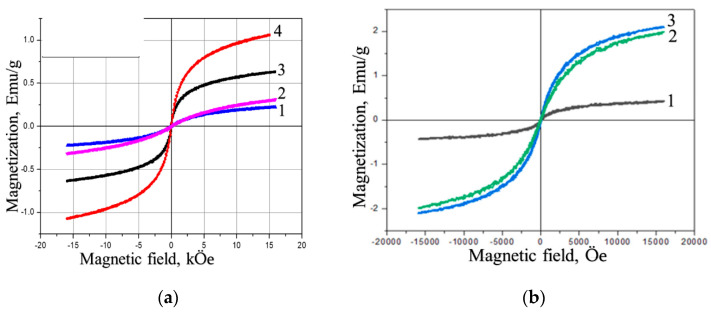
Magnetization curves of the composites at 300 °K. (**a**) I (1), II (2), III (3) and IV (4); (**b**) Ipa (1), IIIpa (2) and IVpa (3).

The magnetization curves for the initial HYAL and four Fe-containing binary composites (Figure 7a) indicate the ferromagnetic nature of the composites. The magnetostatic characteristics of the binary composites—saturation magnetization (Is), residual magnetization (Ir) and coercive force (Hc), calculated from the curves in Figure 7a—are presented in Table 5. The data in Table 5 show that increasing the iron oxide content in the binary composites results in elevating the saturation magnetization from 0.15 for I to 1.1 emu/g for IV but has no effect on the residual magnetization and coercive force. 

As to the magnetization of the ternary HYAL-PA-maghemite composites (Figure 7B), we see a pronounced increase in both saturation magnetization and residual magnetization values with rising iron oxide content and a slight increase in the coercive force (Table 5). In all cases, the magnetostatic characteristics for the ternary composites are higher than for the binary ones. 

The magnetic measurements for dry composites were supplemented by the study of the magnetic susceptibility for the composite microgels in an aqueous solution. First, the HYAL-γ-Fe_2_O_3_ binary composite IV with η = 26.8 wt% was dispersed in bi-distilled water so that 2 wt% composite solution was obtained. The solution was placed in a vessel, from which it entered the peristaltic pump through a silicone hose with a built-in glass capillary, 0.88 mm in diameter. The flow rate was changed from 0.17 to 1.84 cm/s, which formally corresponds to the rate of venous blood in a human body [58]. The microgel particles were stopped by a permanent neodymium magnet, which created a magnetic flux density of 400 T. Then, the experiment was carried out again with the η = 38 wt% HYAL-PA-γ-Fe_2_O_3_ ternary composite IVpa as described above. Thus, in the experiments the binary and ternary composites were used, each with the maximum content of iron oxide nanoparticles.

**Figure 8:**
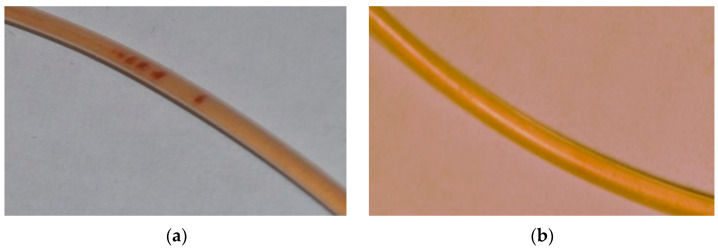
(**a**) Microgel collected with the magnet on the inner surface of capillary. (**b**) No effect of the magnet on the microgel circulation. Composite IV (**a**) and composite IVpa (**b**); flow rate 18.4 cm/s, magnetic flux density 400 T.

The experiment gave a positive result if the magnet stopped the microgel circulation, which was visually controlled by formation of dark spots on the capillary wall as shown in Figure 8a. A negative result was obtained if the magnet could not stop the microgel and form spots on the capillary wall (Figure 8b). The results for different flow rates are collected in Table 6, where positive results are marked with “+” and negative results with “−”.

As follows from the data in Table 6, the binary HYAL-maghemite microgels and the ternary HYAL-PA-maghemite microgels, circulated in the aqueous solution, reacted differently to the permanent magnet. The first, with a lower magnetic susceptibility, could be stopped with the magnet only at a low rate of circulation, 0.37 cm/s or less. On the contrary, the second, with a much higher magnetic susceptibility, were more sensitive to the permanent magnet; these microgels can be stopped with the magnet even at the highest circulation rate, 1.84 cm/s, comparable to the rate of venous blood flow [59]. Thus, by varying the content of iron oxide nanoparticles in the composite (microgel), one can control the magnetic properties of the composites and their sensitivity to the outer magnetic field. 

After removing the magnet, the dark spots shown in Figure 8a disappeared, and the microgels were again distributed throughout the solution. The size of the reconstructed microgels was close to the size of the original microgels before application of an external magnetic field (“magnetic stress”).

### 3.4. Antitumor Activity of Doxorubicin-Loaded Polymer–Maghemite Composites

The magnetic composites are of interest for the controlled delivery of drugs. For the antitumor antibiotic doxorubicin (Dox), the target is nuclear DNA [60]. In our work, Dox was incorporated into two magnetic composites, the binary composite III and the ternary composite IIpa, via mixing of aqueous solutions of the composite and Dox at a carboxylic polymer group-to-Dox molar ratio of Z = [-COOH]/[Dox] = 10 for each case. 

The biological activity of the initial composites, Dox and composites with incorporated Dox was tested towards human breast adenocarcinoma MCF-7 cells using a conventional MTT test, which allowed for quantifying a percentage of surviving cells [61]. As a measure of cytotoxicity, a concentration of the tested sample was used, which caused a 50% death of cells (IC50). The IC50 was calculated in terms of the carboxylic group concentrations for composite III and composite IIpa (column 2 in Table 7), and the Dox concentrations for the Dox alone and for the composites with incorporated Dox, III-Dox and IIpa-Dox (column 3). This allows each component, the polymer and Dox, to contribute to the cytotoxicity of the Dox-containing composites. 

Neither of the polymer composites, the binary III and the ternary IIpa, exhibited cytotoxicity over the entire range of studied composite concentrations up to 1 mM, while individual Dox showed IC50 = 1.06 μM, which correlated with previously described data [62]. Incorporation of Dox in the composites had no effect on its cytotoxicity; they remained at the level of cytotoxicity of Dox itself. In other words, the cytotoxicity of the composites is determined by the cytotoxicity of the antitumor antibiotic. The polymer–iron oxide composites serve as neutral matrixes for encapsulation and transportation of Dox and did not contribute to the total cytotoxicity of the resulting Dox-containing magnetic constructs. Additionally, it may be speculated that the neutral polymer matrix protects the encapsulated drug from undesirable rapid destruction and loss of medicinal properties. 

An important question concerns a possible release of Dox from the Dox-containing magnetic constructs during the magnetic stress experiment, as described in Section 3.3. The Dox release, if it occurs, is accompanied by a change in the composition of the dosage form and most likely its therapeutic activity. The control of the release was performed using the procedure described in Section 3.3. The solution of Dox-loaded magnetic composite was passed with the peristaltic pump through a glass capillary, and the microgel particles were stopped by a permanent neodymium magnet. The solution was poured from the system into a vessel, and the optical density was measured at two wavelengths, 234 nm (D_234_) and 480 nm (D_480_), which corresponded to the two maxima in a Dox UV spectrum [63]. In this prepared solution, D_234_ = 0.071 and D_480_ = 0.014, which is comparable to the corresponding indicators for the solvent, bi-distilled water. For comparison, the solution of the initial Dox-loaded magnetic composite gave D_234_ = 0.65 and D_480_ = 0.21. These results show that the Dox-loaded magnetic composite did not lose Dox during the magnetic stress experiment. 

### 3.5. Enzyme-Induced Degradation of the Polymer–Maghemite Composites

**Figure 9:**
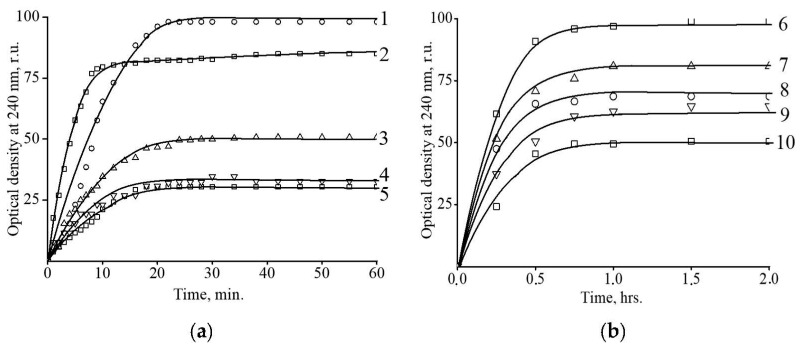
(**a**) Kinetics of hyaluronidase-induced degradation of HYAL (1, control) and the binary composites: I (2), II (3), III (4) and IV (5). (**b**) Kinetics of hyaluronidase-induced degradation of HYAL (6, control) and the ternary composites: Ipa (7), IpaI (8), IIIpa (9) and IVpa (10). Hyaluronidase conc. 1 μg/mL, HYAL conc. 1 mg/mL, pH 7.4, 37 °C.

Eventually, after delivering encapsulated bioactive compounds to the target organs and cells, the Fe-containing microgel carriers should be degraded to smaller particles. The microgels, being composed of sodium hyaluronate, should biodegrade in the presence of a specific hydrolytic enzyme, hyaluronidase [64]. This enzyme cleaves the hyaluronate matrix into short fragments of uronic acid, which can be registered spectrophotometrically by measuring the optical density of the solution at 240 nm. Figure 9a shows the optical density vs. time plots for the four binary HYAL-maghemite microgels with different iron oxide content. The optical density is presented in relative units, while the maximum value for HYAL was taken as 100%. Similar experiments were carried out for the ternary HYAL-PA-maghemite microgels (Figure 9b). 

In all cases, the optical density of the solutions first increased, reached its maximum value after 20–30 min and then did not change. In follows that from there, it took no more than 30 min for degradation to complete. At the same time, the final optical densities Amax for the composites was dependent on the iron oxide content: the higher the η value in the composite, the lower the Amax. More iron oxide required more carboxylic HYAL groups for the cross-linking and formation of the binary and ternary composites. Apparently, only the residual free carboxylic groups could be involved in the enzymatic hydrolysis. This mechanism explains the fall in Amax with the rise in η. 

**Figure 10:**
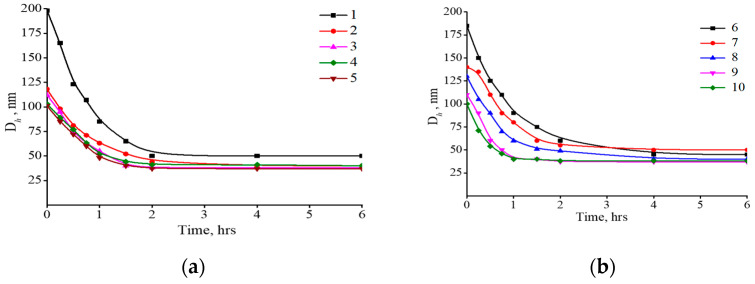
(**a**) Kinetics of hyaluronidase-induced degradation of HYAL (1, control) and the binary composites: I (2), II (3), III (4) and IV (5). (**b**) Kinetics of hyaluronidase-induced degradation of HYAL (6, control) and the ternary composites: Ipa (7), IIpa (8), IIIpa (9) and IVpa (10). Kinetics was controlled by measuring size of polymer and composite particles using dynamic light scattering. Hyaluronidase conc. 1 μg/mL, HYAL conc. 1 mg/mL, pH 7.4, 37 °C.

Additionally, the enzyme-mediated degradation of the composite particles was followed by monitoring of their size (Figure 10). Addition of hyaluronidase to an aqueous solution of HYAL and composites induced a decrease in the size of particles in the system. The degradation developed to a particle size of approx. 40–60 nm, after which the size stopped changing. The minimum size was reached within 1 to 3 h. Thus, the size continued to decrease even after the enzyme had completed hydrolysis of HYAL, which indicates long-term structural rearrangements in the composites after they were attacked by the enzyme. The degraded 40–60 nm particles can be excreted from the body through the kidneys [65].

### 3.6. Enzyme-Induced Release of Doxorubicin from the Polymer–Maghemite Composites 

**Figure 11:**
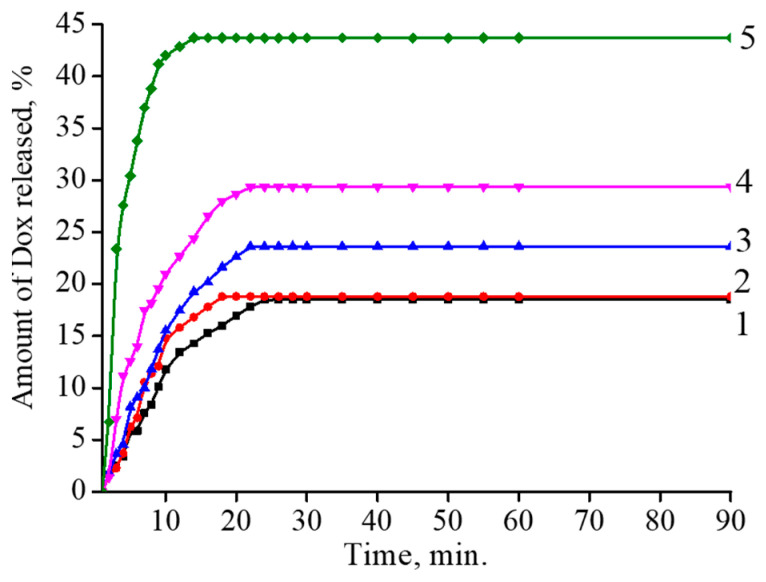
Kinetics of hyaluronidase-induced increase in the fluorescence of Dox incorporated in HYAL (1) and the binary composites: I (2), II (3), III (4) and IV (5). Hyaluronidase conc. 1 μg/mL, HYAL conc. 0.1 mg/mL, [-COOH]/[Dox] = 5, pH 7.4, 37 °C.

Finally, the release of Dox from the magnetic composites after their attack by the enzyme was studied via measuring the intensity of Dox fluorescence. It has been shown that concentration of Dox molecules within a small volume, e.g., in bilayer lipid vesicles (liposomes) or polymer coils, is accompanied by the quenching of Dox fluorescence. Further release of Dox to the surrounding solution restores the fluorescence [66]. Figure 11 presents kinetic curves for hyaluronidase-induced increases in the fluorescence of Dox incorporated in the binary composites. For all the composites, the ultimate level of fluorescence was reached within 20–30 min; this level increased with a rise in the iron content in the composite. This observation was consistent with the data in Figure 10, which show a hyaluronidase-induced decrease in the size of the magnetic composites with elevations in iron content. Thus, an increase in the iron content favored the degradation of the composites and the release of encapsulated Dox. Similar regularities were obtained for the ternary composite with encapsulated Dox.

## 4. Conclusions

A series of Fe-containing polymer composites were synthesized via cross-linking of HYAL or the HYAL/PA mixture with γ-Fe_2_O_3_ nanoparticles. The cross-linking imparted magnetic properties to the composites, more pronounced for the ternary HYAL/PA-γ-Fe_2_O_3_ composites compared with the binary HYAL-γ-Fe_2_O_3_. The composites were dispersed in water and produced microsized hydrogel particles. The ternary HYAL/PA- γ-Fe_2_O_3_ microgels were put into circulation with a peristaltic pump at a speed up to 1.84 cm/s, comparable to the rate of venous blood flow. The circulation was stopped by using a permanent outer magnet with a magnetic flux density of 400 T. Cationic antitumor Dox was electrostatically complexed with the anionic binary HYAL-γ-Fe_2_O_3_ and ternary (HYAL/PA-γ-Fe_2_O_3_ magnetic composite hydrogels in which the Dox showed a cytotoxicity to tumor cells comparable to the cytotoxicity of Dox by itself. The addition of the hydrolytic enzyme hyaluronidase induced degradation of the binary and ternary microgels down to smaller particles and the release of Dox molecules. These results are useful for the preparation of magnetically controlled biodegradable polymer carriers for the encapsulation of bioactive substances.

## Figures and Tables

**Table 1:** The iron content η in the binary composites studied.

No	[HYAL]/[Fe^2+^] Ratio in Reaction Mixture	η, wt%
I	4/1	5.12
II	2/1	14.0
III	1/1	17.0
IV	1/2	26.8

**Table 2:** Hydrodynamic diameter and electrophoretic mobility of the binary composites.

Sample	Fe Content, wt%	D_h_ in Bi-Distilled Water, nm	D_h_ in 0.15 M NaCl Aqueous Solution, nm	EPM in pH 7.0 TRIS Buffer, (μm/s)/(V/cm)
HYAL	0	275	160	−4.23
I	5.12	265	126	−3.21
II	14.0	225	130	−3.21
III	17.9	200	111	−2.85
IV	26.8	150	110	−1.68

**Table 3:** Hydrodynamic characteristics of the binary composites.

Sample	Fe Content, wt%	M_w_ × 10^−6^, Da	R_g_, nm	R*_h_*, nm	R_g_/R*_h_*	2A_2_ × 10^−5^, Mole cm^3^ g^−2^
I	5.12	100	79	180	0.44	1.35
II	14.0	180	67	150	0.45	1.18
III	17.0	220	59	125	0.47	1.15
IV	26.8	250	55	85	0.64	0.9

**Table 4:** The iron content in the ternary composites studied.

№	HYAL/PA Ratio in Reaction Mixture, wt/wt Ratio	η, wt%
Ipa	10/0.5	15.8
IIpa	10/1	17.2
IIIpa	10/2	36.4
IVpa	10/3	38.0

**Table 5:** Magnetostatic characteristics of the Fe-containing composites.

Magnetostatic Characteristic	Binary Composites				Ternary Composites			
	I	II	III	IV	Ipa	IIpa	IIIpa	IVpa
Is, emu/g	0.15	0.25	0.6	1.1	0.3	0.31	1.48	1.84
Ir, emu/g	0.05	0.06	0.06	0.06	0.01	0.03	0.25	0.29
Hc, Э	0.3	0.3	0.3	0.3	0.3	0.5	0.5	0.5

**Table 6:** Ability of the magnet to stop the microgel circulation in an aqueous solution. Stop marked with “+”, no stop with “−”.

Flow Rate, cm/s	Composite IV	Composite IVpa
0.17	+	+
0.37	+	+
0.62	−	+
1.37	−	+
1.84	−	+

**Table 7:** Cytotoxicity of Dox and Dox-loaded magnetic composites towards MCF-7 cells.

Sample	IC_50_, μM	
	Carboxylic Groups	Dox
III	>1000	n/a
IIpa	>1000	n/a
Dox	n/a	1.06 ± 0.05
III-Dox	n/a	1.03 ± 0.04
IIpa-Dox	n/a	1.05 ± 0.04

## Data Availability

Not applicable.

## Supplementary Materials

The following supporting information can be downloaded at https://www.mdpi.com/article/10.3390/polym15173644/s1.
