# Supplementary material for: Magnetically Controlled Hyaluronic Acid–Maghemite Nanocomposites with Embedded Doxorubicin
Source: Polymers (Basel). 2023 Sep 4;15(17):3644. doi: 10.3390/polym15173644 (PMC10489843; doi:10.3390/polym15173644)
Supplement: Supplementary file 1 [file polymers-15-03644-s001.zip › polymers-2557082-supplementary.pdf]

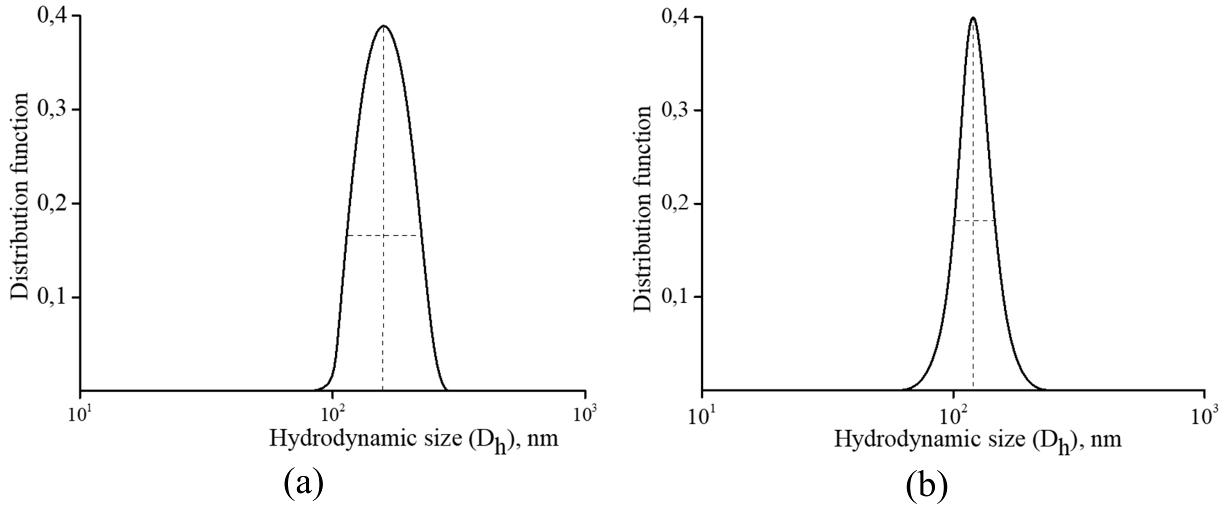

Figure S1. Distribution functions: (a) Initial HYAL and (b) composite (I) in physiological (0.15M NaCl) solution.

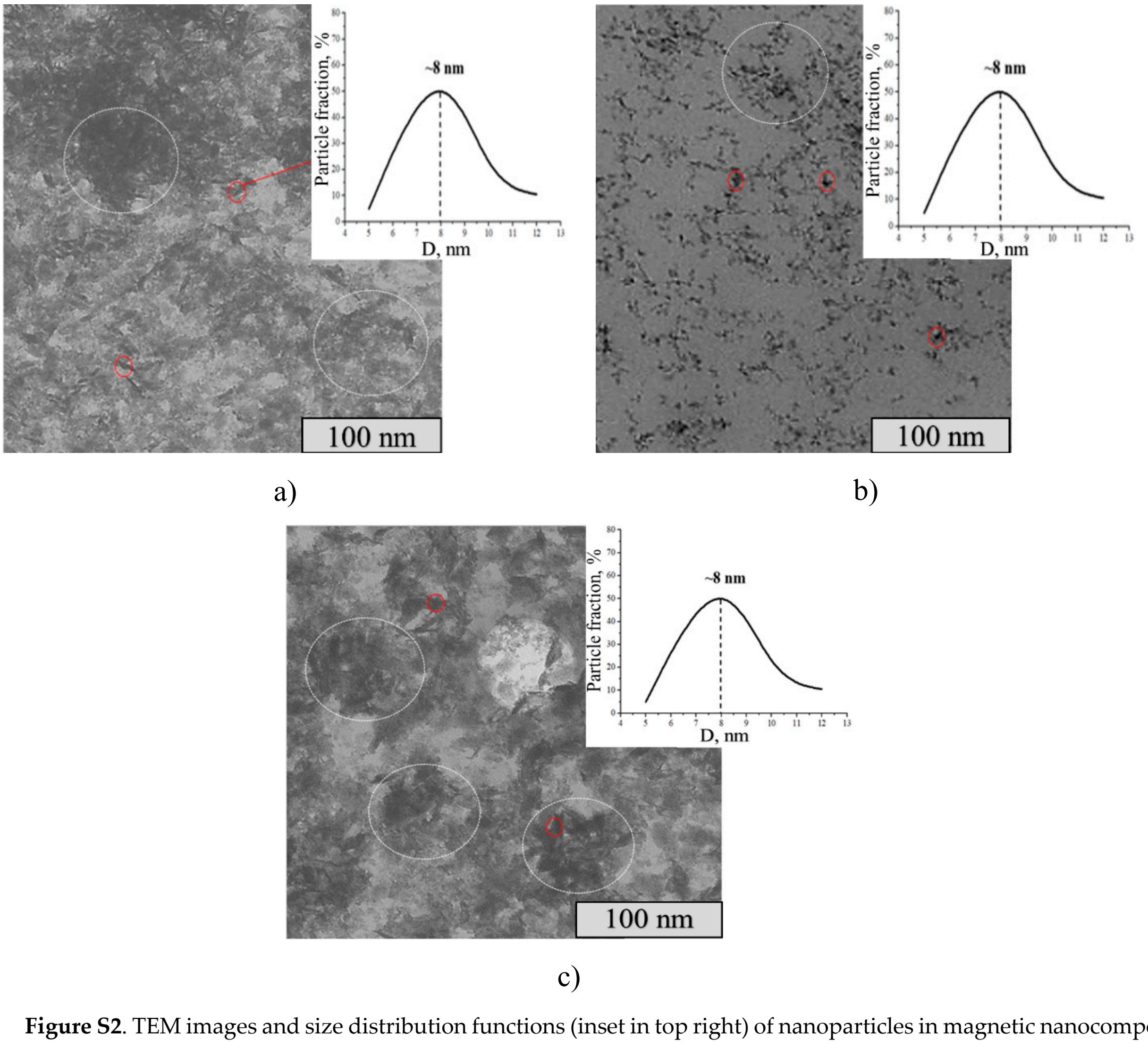

Figure S2. TEM images and size distribution functions (inset in top right) of nanoparticles in magnetic nanocomposites containing iron oxide: **a**) 5.12 % wt. (composite I); **b**) 14.0 % wt. (composite II) and **c**) 17.9% wt. (composite III).
